# Anticancer activities of natural abietic acid

**DOI:** 10.3389/fphar.2024.1392203

**Published:** 2024-04-03

**Authors:** Bashir Ahmad, Chuan Tian, Ji-Xin Tang, John Sieh Dumbuya, Wen Li, Jun Lu

**Affiliations:** ^1^ Department of Pediatrics, Affiliated Hospital of Guangdong Medical University, Zhanjiang, China; ^2^ Guangdong Provincial Key Laboratory of Autophagy and Chronic Non-Communicable Diseases, Institute of Nephrology, Affiliated Hospital of Guangdong Medical University, Zhanjiang, Guangdong, China

**Keywords:** Abietic acid, *Pinus palustris*, natural product, cancer, *Pimenta racemose var. grissea*

## Abstract

Cancer is the main cause of death in the world. There are several therapies that are in practice for cancer cure including radiotherapy, chemotherapy, and surgery. Among the chemotherapies, natural products are considered comparable safe, easily available and cost effective. Approximately 60% of cancer approved FDA drugs are natural products including vinblastine, doxorubicin, and paclitaxel. These natural products have complex structures due to which they work against cancer through different molecular pathways, STAT3, NF-kB, PI3K/AKT/mTOR, cell cycle arrest, mitochondrial dependent pathway, extrinsic apoptosis pathway, autophagy, mitophagy and ferroptosis. AA is a natural abietane diterpenoid compound from *Pinus palustris* and *Pimenta racemose var. grissea* with different pharmacological activities including anti-inflammatory, anti-convulsant, anti-obesity and anti-allergic. Recently it has been reported with its anticancer activities through different molecular mechanisms including NF-kB, PI3K/AKT, call cycle arrest at G0/G1 phase, mitochondrial dependent pathway, extrinsic apoptosis pathway, AMPK pathway and ferroptosis pathways. The literature survey reveals that there is no review on AA anticancer molecular mechanisms, therefore in current review, we summarize the anticancer molecular mechanisms of AA.

## 1 Introduction

Around the world, the second main cause of death is cancer (Pengyu and Lijuan, 2018; Ahmad et al., 2019a; Pengyu Su et al., 2020; Su et al., 2020). According to a World Health Organization (WHO) report, cancer is the cause of more death in the world compared to strokes and coronary heart diseases (Organization, 2015). Aging and overpopulation are the two main causes for increase in cancer (Torre et al., 2015). The global demographic and epidemiologic transition reveals that the cancer is expected to increase in the next decade, especially in low and middle income countries (Bray, 2014). According to an estimation, 18.1 million (m) new cases excluding non-melanoma skin cancer (17 m) 9.8 m cancer related deaths excluding 9.5 m cases of non-melanoma skin cancer, were reported in 2018. When considering both sexes, lung cancer was the most prevalent, accounting for 11.6% of all cases, closely followed by breast cancer in women, also at 11.6%. Prostate and colorectal cancers were next, with incidences of 7.1% and 6.1% respectively. In terms of mortality, lung cancer was the deadliest, responsible for 18.4% of all cancer-related deaths. Colorectal cancer was the second leading cause of death at 9.2%, with stomach and liver cancers each accounting for 8.2% of total cancer fatalities (Bray et al., 2018).

In cancer treatment, the main treatment options are radiotherapy, chemotherapy, and surgery (Qi et al., 2010). These conventional clinical therapies shows limited success in cancer treatment due to the secondary resistance showed by tumors due to different molecular mechanisms (Pengyu and Lijuan, 2018; Ahmad et al., 2019b). Additionally, these therapies ae also costly and show toxicity to normal tissues. In this scenario, search for less toxic, efficient, and cost effective treatment is the need of time (Pengyu and Lijuan, 2018; Ahmad et al., 2019b). Plants derived natural products are currently considered ideal treatment option for cancer treatment due to its low level toxicity, overcome on resistance, easily available and cost-effective (Pengyu and Lijuan, 2018; Ahmad et al., 2020; Jalal et al., 2020). Additionally, almost 60% of FDA approved anti-tumor drugs for clinical use are plant derived (Sanders et al., 2016), for example, vinblastine, doxorubicin, and paclitaxel. These are the well-known anti-tumor drugs which are used in clinical treatment for cancer. (Newman and Cragg, 2016). These natural product have complex structures due to which they work against cancer through different molecular pathways. The reported pathways which natural products regulate in cancer include NF-kB, inflammation, autophagy, PI3K/AKT/mTOR, MEK-ERK, apoptosis and oxidative stress (Ahmad et al., 2020). AA is a natural abietane diterpenoid compound from *Pimenta racemose var. grissea* with different pharmacological activities including anti-inflammatory, anti-convulsant, anti-obesity and anti-allergic (Hwang et al., 2011; Gao et al., 2016; Kang et al., 2018). Recently, the anticancer activities of AA have been reported in different cancers *in vitro* and *in vivo* with overcome on Taxol toxicity (Yoshida et al., 2008; Hsieh et al., 2015; Xu et al., 2017). According to the available literature, AA is new reported compound with anti-cancer activities, therefore, the current review aims to summarize the anticancer studies on different molecular mechanisms of AA.

## 2 Anticancer activities of AA

### 2.1 Cell cycle arrest

The process of cell growth regulation during the cell cycle involves the coordination of specific cyclins with their corresponding cyclin-dependent kinases (CDKs) to form functional complexes, which operate at distinct checkpoints (Lim and Kaldis, 2013). These complexes enable cells to transition from one phase of the cell cycle to the next. This regulatory mechanism ensures proper cell growth and division and helps to prevent errors from occurring. Ultimately, the cell cycle progresses until it is ready to enter a new phase (Lu et al., 2006). Cell cycle progression is controlled by the activity of CDKs, which are negatively regulated by specific CDK inhibitors. When the regulation of these cell cycle checkpoints fails, it can lead to genomic instability, DNA damage, and mutations, ultimately resulting in genetic disturbances and potentially giving rise to cancer (Yang et al., 2010). It is critical for the cell cycle to be carefully controlled and for the checkpoints to function properly to maintain the integrity of the genetic material and prevent the development of cancer (Yang et al., 2010).

**FIGURE 1:**
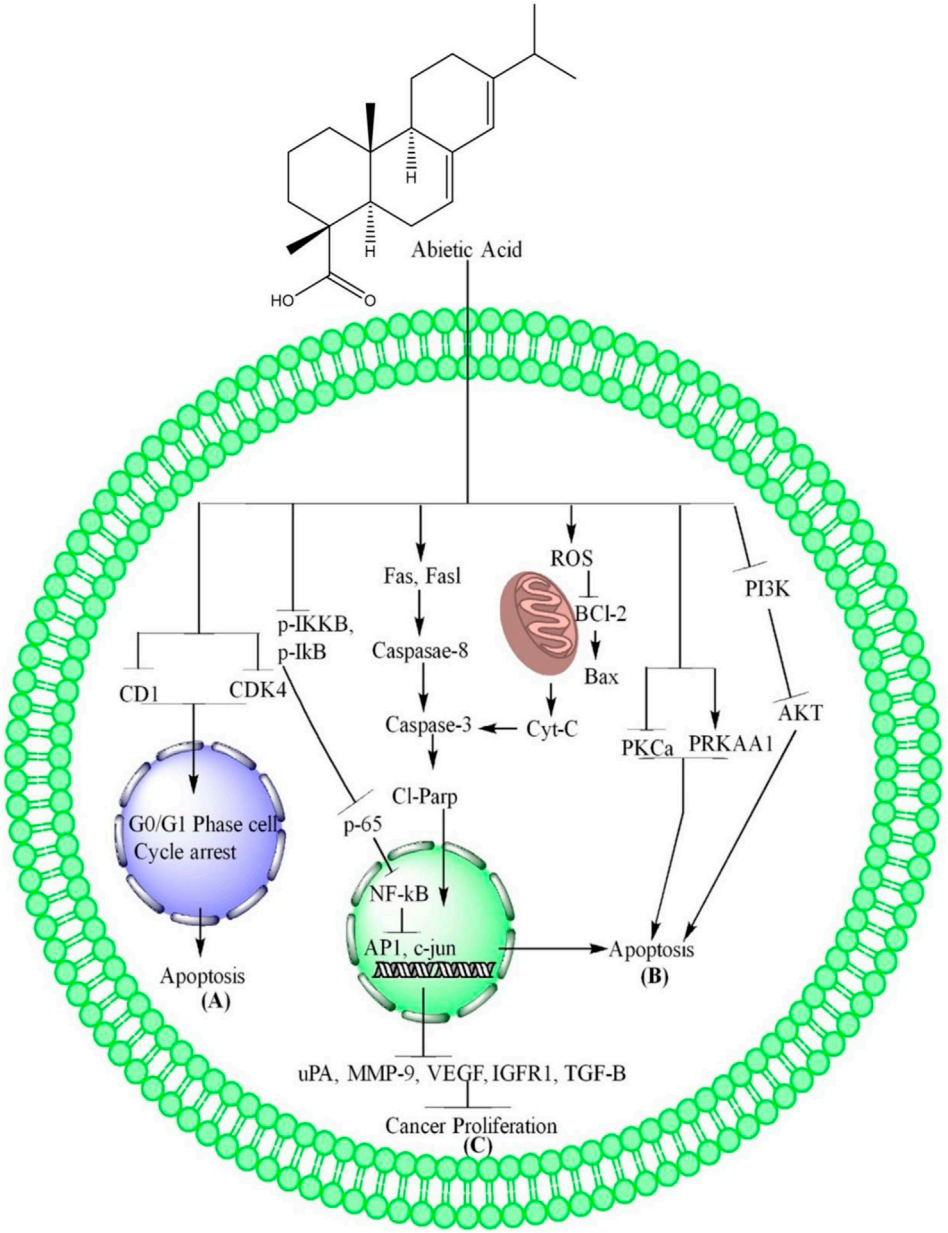
AA induces apoptosis and inhibits proliferation of cancer. **(A)** AA inhibits CD1 and CDK4, resulting to apoptosis through G0/G1 phase cell cycle arrest. **(B)** AA increase ROS level in cancer cells which further cause oxidative stress due to which the Bcl-2 downregulates and upregulate Bax, increase the release of Cyt-c, the cyt-c causes the upregulation of caspase-3, cleave parp which enter nucleus where they cause DNA damage and induces mitochondrial dependent apoptosis. AA also causes extrinsic apoptosis in cancer cells through upregulation of Fas, Fasl, caspase-8 which further upregulate the Cl-Parp. In addition, AA induces apoptosis in cancer cells through AMPK via inhibition of PKCa, PRKAA1 and PI3K/AKT/mTOR pathways via inhibition of PI3K and AKT phosphorylation. **(C)** AA inhibits the cancer cells proliferation through inhibition of IKKβ and IkB phosphorylation which further inhibit the NF-kB translocation into nucleus due to which AP1 and c-Jun are downregulation. The downregulation of these genes further inhibits the expression of uPA, MMP-9, VEGF IGFR1, TGF-β and lead to inhibition of cancer cells proliferation.

AA, a compound derived from *Pinus palustris*, showed promising anti-proliferative activity against the human breast cancer cell line MCF-7 through causing G2/M cell arrest and subG0 -G1 phase cell cycle arrest. The AA caused cell cycle arrest and induction of apoptosis in MCF-7 cells (Haffez et al., 2022). AA impacted the cell cycle distribution of NSCLC, PC-9 and H1975 cells by arresting them at the G0/G1 phase. The expression of cell cycle-related proteins, such as cyclin D1 and cdk4, was also downregulated by AA, suggesting that it effectively arrested the cells in the G0/G1 phase through reducing the expression of these proteins (Liu et al., 2019). The summarized form of AA induced cells cycle arrest is depicted in Figure 1A and Table 1.

**TABLE 1:** Anticancer effect of AA through different pathways.

Pathway	Effect of AA	Upregulated genes	Downregulated genes	Cell/Model organism	Reference
**Cell Cycle Arrest**	AA caused G2/M cell arrest and subG0 -G1 phase cell cycle arrest in MCF-7 cells. It also arrested NSCLC, PC-9 and H1975 cells at the G0/G1 phase	-	Cyclin D1, cdk4	MCF-7, NSCLC, PC-9, H1975	Haffez et al. (2022)
					Liu et al. (2019)
**Mitochondrial Pathway**	AA downregulated the Bcl-2 and upregulated the Bax, released Cyt-c from mitochondria, increased the expression of cleaved parp, and resulted in apoptosis in MCF-7 and NSCLC cells	Bax, Cleaved parp	Bcl-2	MCF-7, NSCLC	Haffez et al. (2022)
					Liu et al. (2019)
**Extrinsic Apoptotic Pathway (EAP)**	AA regulated the EAP through overexpression of apoptotic genes Fas, Fasl which further increased the expression of caspase-8, leading to activation of caspase-3 and apoptosis	Fas, Fasl, Caspase-8, Caspase-3	-	MCF-7	Haffez et al. (2022)
**AMPK Pathway**	AA downregulated PKC-a levels while overexpressing PRKAA1, a key kinase in MCF-7 resistance and metastasis cells. This overexpression activated AMPK.	PRKAA1	PKC-a	MCF-7	Haffez et al. (2022)
**PI3K/Akt, ERK Pathways**	AA caused a gradual decrease in PI3K protein levels and significantly inhibited Akt activation in a dose-dependent manner. There was no significant influence on phosphorylated p38 or phosphorylated ERK1/2	-	PI3K, Akt	Not specified	Hsieh et al. (2015)
**Nuclear Factor Kappa B (NF-kB) Pathway**	In NSCLC, AA downregulated the TNF-α induced activation of IKKβ, IkB and blocked the NF-kB nuclear translocation dose-dependently. AA directly bound to IKKβ, while its binding with IKKα was much lower, suggesting specificity for IKKβ. In addition, AA downregulated the proliferation (VEGF, IGFR1, TGF-β) and oncogenic genes (C-myc and NF-κB), and increased levels of antioxidants	-	IKKβ, IkB, VEGF, IGFR1, TGF-β, C-myc, NF-κB	NSCLC	Liu et al. (2019)
					Haffez et al. (2022)
**Ferroptosis in Cancer**	AA has a selective effect on inhibiting the viability of bladder cancer (BC) cell lines (J82, T-24, 5637) in a time and dose dependent manner. It has little effect on the normal urothelial cell line SV-HUC-1. AA induces ferroptosis in BC cells through upregulation of GPX4 which further affects the MDA, iron and GSH level in BC cells. AA treatment reduced lung metastasis of B16F10 cells in mice, resulting in lower mean lung weight and fewer countable nodules in the lungs compared to the control group. AA effectively reduced tumor size in mice with breast cancer xenografts, without affecting the mice’s body weight	GPX4, HMOX1, Nrf2, DDT3, ATF4, SOD1, HSPB1, P53, XBP1, catalase, GRX1, HO-1	-	BC (J82, T-24, 5637), SV-HUC-1, B16F10 (mice), Breast cancer xenografts (mice)	Xu et al. (2023)

### 2.2 AA regulate mitochondrial pathway

In induction of apoptosis, the mitochondrial dependent pathway plays a crucial role, and its disruption can prevent apoptosis from occurring. The Bcl-2 family proteins regulate this pathway by modulating the mitochondrial membrane permeability, which determines the release of different apoptotic proteins from mitochondria like cytochrome c (Cyt-c) (Wu and Bratton, 2013). BclxL and Bcl-2 are the anti-apoptotic proteins which promote cell survival by preventing apoptosis, while pro-apoptotic proteins such as BAX induce apoptosis by creating mitochondrial stress (Reed, 2006). Plant-derived compounds are viewed as a safe and cost-effective approach to targeting cancer through various pathways, including the mitochondrial-dependent pathway. These compounds act through multiple mechanisms including mitochondrial dependent pathway, to induce apoptosis in cancer cells, making them a promising therapeutic option (Ahmad et al., 2019b; Ahmad and Gamallat, 2021).

In breast cancer MCF-7 and NSCLC cells, AA downregulated Bcl-2 and upregulated Bax, resulting in the release of Cyt-c from mitochondria and increasing the expression of cleaved PARP, which ultimately led to apoptosis (Liu et al., 2019; Haffez et al., 2022). The AA mechanisms are further summarized in Figure 1B and Table 1.

### 2.3 AA and extrinsic apoptotic pathway (EAP)

Proteins from the Tumor Necrosis Factor (TNF) family, including Fas or TNF-receptor-1 (TNFR-1), activate the extrinsic apoptosis pathway. This pathway is an important mechanism for inducing programmed cell death (Locksley et al., 2001). TNFR-1 or Fas proteins through Fas associated death domains activate the caspase-8, which further activate the caspase-3 and help in cells apoptosis (Ashkenazi, 2008; Fulda, 2015). Natural products regulate this pathway in different cancers (Fatehchand et al., 2017; Kang et al., 2017). AA is a natural product derived from medicinal plants.

The AA regulate the EAP through overexpression of apoptotic genes Fas, Fasl which further increase the expression of caspase-8, caspase-8 results in activation of caspase-3 and led the cells to apoptosis (Haffez et al., 2022). These mechanisms of AA are further summarized in Figure 1B and Table 1.

### 2.4 AA and AMPK pathway

Protein kinase C (PKC) has been traditionally viewed as an oncoprotein, meaning that it was believed help in progression of cancer cells growth and development (Newton, 2018). PRKAA1, is the subunit of AMP activated protein kinases (AMPK), which play an important role in control of cellular metabolism through its phosphorylation. Recent studies have found that genetic variations in PRKAA1 have a close link with gastric cancer. These findings suggest that PRKAA1 and AMPK may play a significant role in gastric cancer progression and development (Zhang et al., 2020) which make it an important therapeutic target in cancer treatment.

AA has been shown to downregulate PKC-a levels while overexpressing PRKAA1, a key kinase in MCF-7 resistance and metastasis cells. This overexpression activates AMPK (Haffez et al., 2022) as shown in Figure 1B and Table 1.

### 2.5 AA and PI3K/Akt, ERK pathways

PI3K/AKT/mTOR pathway plays an important role in regulating cell proliferation, protein synthesis, and apoptosis. Inhibition of this pathway has been shown to have therapeutic potential for various diseases, including cancer (Courtney et al., 2010; Steelman et al., 2011). Understanding the specific mechanisms of activation of this pathway in different types of cancer is important for developing targeted therapies that can inhibit its activity and suppress tumor growth (Samuels et al., 2004; Samuels and Velculescu, 2004; Wong et al., 2010). Plant-derived compounds are a promising approach to treating cancer, as they are generally considered safe and cost-effective. These compounds have been shown to target cancer cells through different pathways, including the PI3K/AKT and ERK pathways, which play crucial roles in regulating cell growth and survival. By targeting these pathways, plant-derived compounds have the potential to inhibit the growth and proliferation of cancer cells, making them a promising avenue for the development of novel cancer therapies (Ahmad and Gamallat, 2021). AA is also a natural product derive from plant.

AA caused a gradual decrease in PI3K protein levels and significantly inhibited Akt activation in a dose-dependent manner. There was no significant influence on phosphorylated p38 or phosphorylated ERK1/2 (Hsieh et al., 2015). These mechanisms of AA are depicted in Figure 1B and Table 1.

### 2.6 AA and nuclear factor kappa B (NF-kB) pathway

The NF-kB is a complex pathway which is consist of five homo and hetero-dimers of Reticuclo-endotheliosis oncogenes cellular (Rel) family including c-Rel, RelA(p65), NF-kB1 (p50/p65), RelB and NF-kB2 (p50/p65) (Sen and Baltimore, 1986). In different cancers the NF-kB pathway become dysregulated (Perkins, 2007) including colon, breast, liver, ovarian, leukemia and lymphoma cancers (Bassères and Baldwin, 2006; Prasad et al., 2010; Arkan and Greten, 2011). As dysregulation of NF-kB pathway is involved in the progression of different cancers, therefore it is a good therapeutic target in treatment of different cancers. Numerous natural substances have been identified as regulators of the NF-kB pathway in various types of cancer (Ahmad et al., 2021; Ahmad and Gamallat, 2021).

In NSCLC, AA downregulates the TNF-α induced activation of IKKβ, IkB and block the NF-kB nuclear translocation dose-dependently. AA directly bound to IKKβ, while its binding with IKKα was much lower, suggesting specificity for IKKβ. The causal relationship between the functional effects and the signaling pathway was supported by the results of overexpression of IKKβ in PC-9 cells, which showed impaired anti-proliferative and apoptosis effects of AA. The authors have also confirmed the binding of AA to IKKβ by surface plasmon resonance (SPR) experiments and the specificity of binding was shown to be higher for IKKβ than IKKα. The study concludes that AA could be a promising lead compound for the discovery of novel IKKβ inhibitors and a potential agent for the treatment of NSCLC (Liu et al., 2019). In addition, AA downregulate the proliferation (VEGF, IGFR1, TGF-β) and oncogenic genes (C-myc and NF-κB), and increased levels of antioxidants (total antioxidant capacity as compared to negative control without (W/O) H_2_O_2_) (Haffez et al., 2022). These mechanisms of AA are further summarized in Figure 1C and Table 1.

### 2.7 AA and ferroptosis in cancer

Ferroptosis is a type of cell death that relies on intracellular iron and differs from other forms of cell death such as apoptosis, necrosis, and autophagy (Zhang et al., 2022). Research suggests that ferroptosis can play a significant role in suppressing tumor growth, which presents an opportunity for cancer therapy (Zhang et al., 2022). However, developing resistance to cancer therapy remains a challenge, and efforts to overcome drug resistance have been the focus of numerous preclinical and clinical studies (Zhang et al., 2022). Interestingly, ferroptosis has been associated with resistance to cancer therapy, and triggering ferroptosis has been shown to reverse drug resistance (Zhang et al., 2022).

AA has a selective effect on inhibiting the viability of bladder cancer (BC) cell lines (J82, T-24, 5637) in a time and dose dependent manner (Xu et al., 2023). It has little effect on the normal urothelial cell line SV-HUC-1 (Xu et al., 2023). Gene expression analysis shows that the ferroptosis pathway and redox signaling pathway are enriched after AA treatment, suggesting AA has selectively antitumor effects against BC (Xu et al., 2023) The type of cell death caused by AA in bladder cancer cells was investigated using various inhibitors (Xu et al., 2023). The apoptosis (z.VAD-FMK, z. VAD) and necrosis inhibitors (Necrostatin-1, Nec) had little effect on the viability of BC cells, while ferroptosis inhibitors and ROS scavengers (N-Acetyl-L-cysteine, NAC) significantly increased it. Cell death assays showed that only ferroptosis inhibitors (Ferrostatin-1, Fer-1; Liproxstatin-1, Lip-1; Deferoxamine, DFO) protected BC cells from AA-induced death. These findings suggest that AA may induce ferroptosis in BC cells (Xu et al., 2023).

#### 2.7.1 Molecular mechanisms of ferroptosis regulated by AA

Ferroptosis is a type of regulated cell death that is distinct from other types of cell death in its morphological, biochemical, and genetic features (Dixon et al., 2012). Ferroptosis is a unique form of cell death triggered by an imbalance in intracellular iron regulation that causes an excess accumulation of toxic lipid reactive oxygen species (ROS). When these ROS overwhelm the cell’s antioxidant defenses, they damage the cell membrane, leading to cell death. This oxidative stress related to lipid peroxidation and iron metabolism is what distinguishes ferroptosis from other forms of cell death (Cao and Dixon, 2016; Stockwell et al., 2017). Ferroptosis can be regulated by the p53 pathway, including mutant p53, but its regulation appears to be highly dependent on the specific cellular context (Liu et al., 2020), the decrease in p53 level have link with decrease in Nuclear factor erythroid (Nrf2) expression (Chen et al., 2012). The role of HO-1 in ferroptosis is complex and may depend on various factors such as the kinetics of its induction, its level of expression, the specific cell type, and experimental conditions (Hassannia et al., 2018). Nrf2 causes activation of HO-1. GPX4, a type of glutathione peroxidase, is crucial in maintaining the balance of oxidative stress and is therefore essential in preventing ferroptosis. GPX4 achieves this by reducing lipid hydroperoxides and preventing the accumulation of lipid peroxidation in cell membranes (Seibt et al., 2019). ATF4 is involved in the proliferation of cancer cells (Du et al., 2021) and show resistance to therapies through inhibition of ferroptosis (Gao et al., 2021). Heme oxygenase-1 (HMOX1) is involved in the cytoprotection, promote cancer metastasis while different drugs including erastrin reverses its resistance by ferroptosis (Liao et al., 2023). These mechanisms reveal that ferroptosis is a new target for cancer therapy through different drugs. Different natural products cause cancer cells death including ferroptosis (Chen et al., 2020).

**FIGURE 2:**
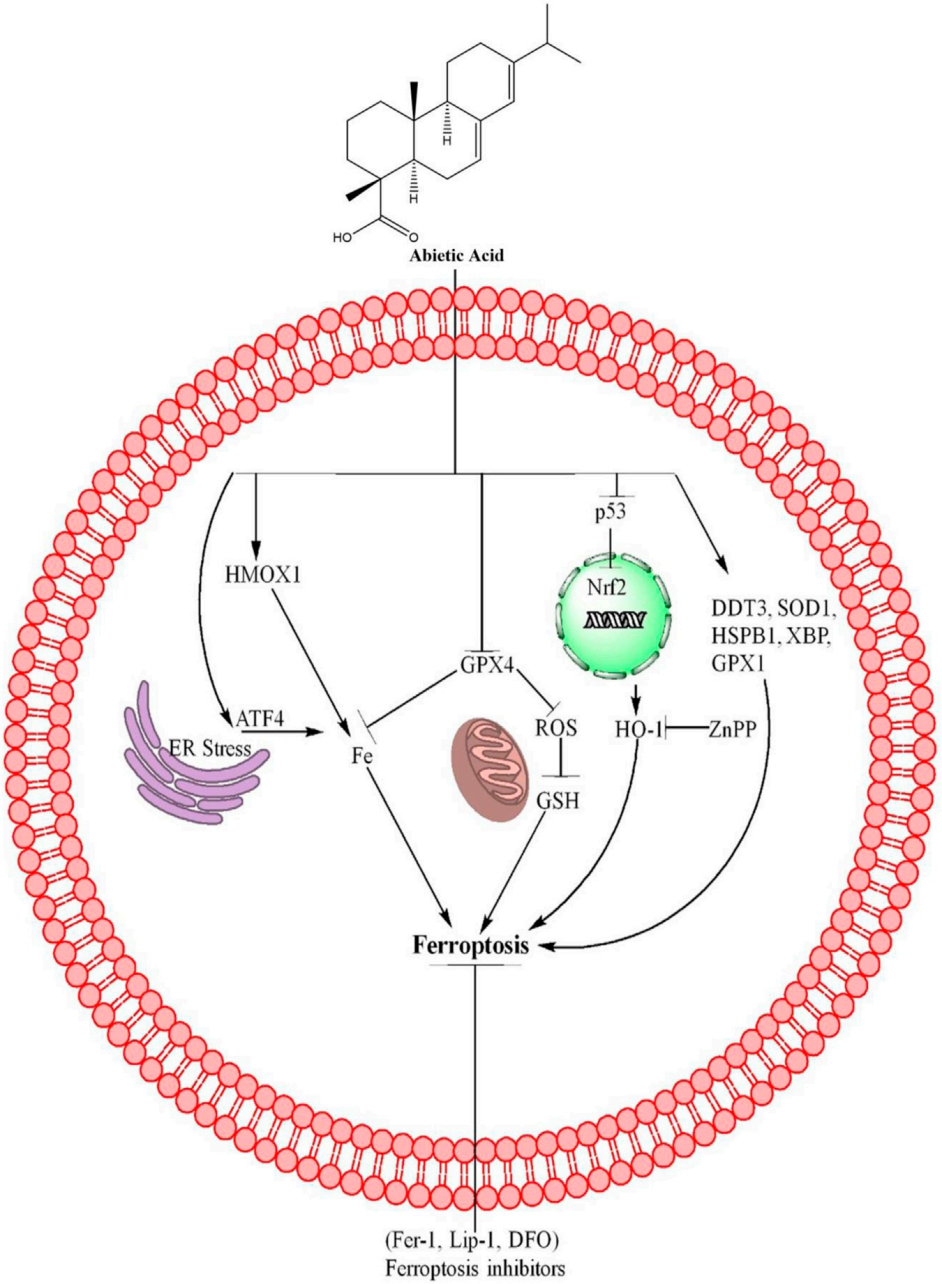
AA causes ferroptosis through different mechanisms. AA inhibits the GPX4 expression which further inhibits the Fe+ accumulation, ROS, and GSH level, resulting ferroptosis in cancer cells. AA also inhibits the p53, Nrf2, which further increases the expression of HO-1 and causes ferroptosis. ZnPP reverses the ferroptosis by inhibiting HO-1 expression. In addition, AA also induces ferroptosis through upregulation of HMOX1 and ATF4. Furthermore, AA upregulates the expression of DDT3, SOD1, HSPB1, XBP and GPX1 and lead to cells death through ferroptosis. The ferroptosis death was reversed by ferroptosis inhibitors including Fer-1, Lip-1 and DFO, revealing that AA induced cell death was ferroptosis.

AA is also a natural compound derived from *P. palustris* (Haffez et al., 2022) and causes cancer cells death through different mechanisms, including ferroptosis. AA induces ferroptosis in BC cells through upregulation of GPX4 which further affects the MDA, iron and GSH level in BC cells. These results suggest that AA induces ferroptosis in BC cells partially through inhibition of GPX4 (Xu et al., 2023). In addition AA upregulate the HMOX1, Nrf2, DDT3, ATF4, SOD1, HSPB1, P53, XBP1, catalase, GRX1 and HO-1 (encoded by HMOX1) (Xu et al., 2023). Knocking down HO-1 with shRNA reduced AA-induced cell death, rescued cell survival, and diminished the effects of AA on ROS, iron, MDA, and GSH levels. These findings suggest that the upregulation of HO-1 is crucial for AA-induced ferroptosis in bladder cancer cells (Xu et al., 2023). The role of HO-1 in ferroptosis induced by AA in BC cells was further confirmed using ZnPP, a specific inhibitor of HO-1. Results showed that ZnPP rescued cell viability and reduced cell death caused by AA. ZnPP also reversed the effects of AA on levels of ROS, iron, MDA, and GSH, further confirming that the increase in HO-1 is crucial for ferroptosis induced by AA (Xu et al., 2023). These mechanisms of AA are further summarized in Figure 2 and Table 1.

### 2.8 Molecular docking

The mechanism of action between AA and IKKβ was shown to be through hydrophobic interactions by molecular docking and molecular dynamics (MD) simulations (Liu et al., 2019). AA has strong binding efficiency with the AchE and HDAC3 receptors, as indicated by Ligplot analysis and root mean square deviation analysis. *In vitro* tests with HeLa cells showed that abietic acid induces apoptosis in a concentration-dependent manner, suggesting its potential as a promising terpenoid for treating Alzheimer’s disease and cervical cancer (Ramnath et al., 2018).

## 3 AA improves taxol activity

The results showed that combining AA with Taxol, a clinical chemotherapy drug, improved the inhibition of cell viability of B16F10 cells after 24 and 48 h of treatment (Hsieh et al., 2015). The combination of Taxol and 50 μM AA reduced the number of viable B16F10 cells to the greatest extent (Hsieh et al., 2015).

## 4 *In Vivo* study

AA treatment reduced lung metastasis of B16F10 cells in mice, resulting in lower mean lung weight and fewer countable nodules in the lungs compared to the control group. Histopathological analysis showed a reduction in tumor mass in the lungs of AA-treated mice. Body weight was not affected by AA treatment (Hsieh et al., 2015). AA effectively reduced tumor size in mice with breast cancer xenografts, without affecting the mice’s body weight. The study also found that AA increased iron and MDA levels in the tumor tissues and decreased GPX4 levels, indicating that it induces ferroptosis and has antitumor effects against breast cancer cells (Xu et al., 2023). These mechanisms of AA are further summarized in Figure 2.

## 5 AA toxicity in human and animals

A study on colophony sensitive patients and guinea pigs reveal that, AA is not a contact allergen (Karlberg et al., 1985). Another study reveals that AA and other plants derived pure secondary compound did not show any toxicity against *Schistocerca americana* (Bernays, 1991). Additionally, the AA shows less genotoxicity in juvenile *Dicentrarchus labrax L* compared to dehydroabietic acid (Gravato and Santos, 2002). AA in its oxidized form (dihydroxy acid) causes irritation in guinea pigs when applied on its skin (Khan and Saeed, 1994). These studies suggest that the AA alone are non-toxic to human or other animals.

## 6 Conclusion and recommendations

The available studies on AA against cancer show that the AA regulates cancer through NF-Kb, PI3K/AKT, G0/G1 phase cell cycle arrest, mitochondrial dependent pathway, extrinsic apoptosis pathway, AMPK pathway and ferroptosis pathways. Additionally, AA is a candidate natural compound that improves the anticancer effect of available drugs like Taxol when used in combination and might be helpful to use the less amount of Taxol or other drug with AA in cancer treatment due to which the Taxol or other anticancer drugs toxicity can be decreased. Additionally, we suggest that to explore the effect of AA *in vitro* and *in vivo* models through different molecular pathways including apoptosis pathways and autophagy pathways. In apoptosis pathways, we further suggest focusing on STAT-3 pathway, Wnt/β-Catenin pathway, endoplasmic reticulum stress mechanisms, and mitogen-activated protein Kinase/extracellular signal-regulated-kinase pathways in apoptosis. In autophagy pathways including PI3K/AKT/mTOR and AMPK/mTOR pathways, Akt, p38 MAPK, ERK1/2, and JNK signaling pathways are warranted to study in future research.

In anticancer research of AA, we further suggest the investigation of AA anticancer effects through different *in silico* tools including Molecular Docking, ADME/Pharmacokinetic Predictions, Virtual Screening, Network Pharmacology Let’s and Systems Biology Analysis. Briefly, *in silico* molecular docking, researchers can predict AA interactions with specific cancer-related proteins. By simulating binding interactions, researchers can identify potential targets and pathways. Assessing the absorption, distribution, metabolism, and excretion (ADME) of AA computationally provides insights into its bioavailability and pharmacokinetics. This information guides drug development. *In silico* screening of AA against databases of cancer-related proteins can identify novel targets. Constructing interaction networks involving AA, cancer-related genes, and pathways can reveal intricate connections. Network-based approaches help uncover hidden relationships. Integrating omics data (genomics, proteomics, etc.) with computational models allows researchers to explore AA’s impact on cancer-related pathways comprehensively.

AA in cancer treatment is in its initial phase for research and clinical trials are not reported. AA can be used for clinical trials, after the exploration of its mechanisms through the above-mentioned pathways and toxicity in different models through methods. In clinical trials we suggest the Phase I trials, Phase II trials, Phase III trials, and Mechanism-based trials. In Phase I trials, the researchers can assess the safety, tolerability of AA on a small group of patients with different types of cancer to evaluate the safety, efficacy and identify the optimal dose for each type of cancer. Once the safety and dosage are established, Phase II trials could be conducted to evaluate the efficacy of AA in a larger group of patients. The primary endpoint could be the response rate or progression-free survival. Following phase II trials, if Phase II trials show that AA is effective, it could then proceed to Phase III trials. These trials would compare the effectiveness of AA against the current standard of care in a large group of patients. The primary endpoint could be overall survival or progression-free survival. AA regulate cancer through different mechanisms, therefore, after successful end of Phase III trials, Mechanism-based trials can be designed to specifically include patients with cancers that are known to be driven by reported pathways.
